# Recent Advances in Sensor–Actuator Hybrid Soft Systems: Core Advantages, Intelligent Applications, and Future Perspectives

**DOI:** 10.1002/advs.202302775

**Published:** 2023-09-26

**Authors:** Chankyu Han, Yongrok Jeong, Junseong Ahn, Taehwan Kim, Jungrak Choi, Ji‐Hwan Ha, Hyunjin Kim, Soon Hyoung Hwang, Sohee Jeon, Jihyeon Ahn, Jin Tae Hong, Jin Joo Kim, Jun‐Ho Jeong, Inkyu Park

**Affiliations:** ^1^ Department of Mechanical Engineering Korea Advanced Institute of Science and Technology (KAIST) Daejeon 34141 Republic of Korea; ^2^ Department of Nano Manufacturing Technology Korea Institute of Machinery and Materials (KIMM) Daejeon 34103 Republic of Korea; ^3^ Radioisotope Research Division Korea Atomic Energy Research Institute (KAERI) Daejeon 34057 Republic of Korea

**Keywords:** integrated devices, intelligent systems, soft actuators, soft sensors, soft systems

## Abstract

The growing demand for soft intelligent systems, which have the potential to be used in a variety of fields such as wearable technology and human‐robot interaction systems, has spurred the development of advanced soft transducers. Among soft systems, sensor–actuator hybrid systems are considered the most promising due to their effective and efficient performance, resulting from the synergistic and complementary interaction between their sensor and actuator components. Recent research on integrated sensor and actuator systems has resulted in a range of conceptual and practical soft systems. This review article provides a comprehensive analysis of recent advances in sensor and actuator integrated systems, which are grouped into three categories based on their primary functions: i) actuator–assisted sensors for intelligent detection, ii) sensor‐assisted actuators for intelligent movement, and iii) sensor‐actuator interactive devices for a hybrid of intelligent detection and movement. In addition, several bottlenecks in current studies are discussed, and prospective outlooks, including potential applications, are presented. This categorization and analysis will pave the way for the advancement and commercialization of sensor and actuator‐integrated systems.

## Graphical abstract

This review highlights the recently demanding soft sensor–actuator hybrid intelligent systems and groups based on their primary functions: i) actuator–assisted sensors for intelligent detection, ii) sensor‐assisted actuators for intelligent movement, and iii) sensor–actuator interactive devices for hybrid of intelligent detection and movement. In addition, several bottlenecks are discussed, and prospective outlooks, including potential applications, are presented.

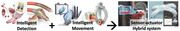

## 1 Introduction

Over the past decade, soft systems comprising one or more soft transducers such as sensors,^[^ ^1^, ^2^, ^3^, ^4^ ^]^ actuators,^[^ ^5^, ^6^, ^7^, ^8^, ^9^ ^]^ heaters,^[^ ^10^ ^]^ and energy harvesters^[^ ^11^ ^]^ have attracted significant attention because of their wide range of applications, including human motion monitoring,^[^ ^12^, ^13^, ^14^, ^15^, ^16^, ^17^ ^]^ structural health monitoring,^[^ ^18^ ^]^ artificial muscles,^[^ ^19^ ^]^ and human–robot interaction systems.^[^ ^20^, ^21^ ^]^ Recently, studies on sensor‐actuator hybrid systems have been actively conducted to exploit the synergistic functions of individual devices, owing to the increasing demand for highly advanced and intelligent systems in soft devices. Generally, they can be categorized into three groups depending on the basic fields and primary functions of soft intelligent systems. First, in the field of soft sensors, diverse types of sensors, such as pressure,^[^ ^22^, ^23^, ^24^, ^25^ ^]^ strain,^[^ ^13^, ^26^ ^]^ and temperature sensors,^[^ ^27^ ^]^ have been developed in soft forms. However, they suffer from positioning issues. Notably, they must be properly attached to the target objects at the desired locations for accurate measurement. However, passive sensors without movable parts or morphable properties have limitations in their applications. If these movable or morphable platforms are added to sensors, accurate data can be acquired from the entire environment without installing sensors in every space or repositioning them. Therefore, actuator‐assisted sensing systems have been proposed to address these issues. Two types of actuators, namely moving and inflating actuators, are frequently used for intelligent detection. For example, movable robots such as walking and flying robots are integrated with soft sensors to automatically reposition the sensors at desired locations.^[^ ^28^, ^29^, ^30^ ^]^ In addition, inflatable actuator‐integrated sensor systems have been developed to facilitate the conformal contact of sensors with target objects after positioning.^[^ ^31^, ^32^, ^33^, ^34^, ^35^ ^]^ Second, numerous soft actuators, such as pneumatic,^[^ ^36^ ^]^ electrothermal,^[^ ^37^ ^]^ and magnetic actuators,^[^ ^38^ ^]^ have been developed; however, they suffer from the inability of precise control of their motion owing to their nonlinear and wide‐range movement and deformable body, which cannot be accurately tracked or can be easily constrained by conventional rigid sensors, requiring the adoption of soft sensors. Therefore, various soft sensors have been integrated with soft actuators to address these limitations. Two types of sensors, namely pressure and strain sensors, are frequently used to realize intelligent movement. For example, if a pressure sensor is integrated onto the tip of a gripper, it can be used to control the contact force when gripping an object.^[^ ^39^ ^]^ In addition, the integration of the soft strain sensor into the robotic arms enables the proprioceptive motions of the actuators.^[^ ^40^, ^41^, ^42^, ^43^, ^44^, ^45^ ^]^ Finally, as ideal soft intelligent systems, sensor, and actuator interactive devices have recently been investigated. In the case of the abovementioned systems, the primary function is focused only on intelligent detection or intelligent movement. However, for efficient execution of more intricate tasks, it is essential to have a combination of intelligent detection and movement achieved through the complementary interaction of sensors and actuators. In a typical scenario involving sensor and actuator integrated systems, the actuator aids the sensor in reaching the target object and ensuring that it makes proper contact. Concurrently, the sensor measures the environmental input and gathers information about the actuator's state, while the actuator is responsible for repositioning the sensor to the desired position or next target.^[^ ^46^, ^47^, ^48^, ^49^ ^]^ In recent times, the field of soft sensor and actuator integrated systems has made noteworthy advancements, revealing diverse conceptual and practical soft systems. Despite the ongoing progress in the research of sensor‐actuator hybrid soft systems, previous literature reviews have predominantly concentrated on individual soft sensors^[^ ^50^, ^51^ ^]^ or soft actuators.^[^ ^52^ ^]^ As a result, there is a need for a comprehensive and detailed review of the latest developments in this area.

This review article aims to fill this gap by summarizing the trends of recent studies in the sensor‐actuator hybrid soft systems, focusing on the working scenarios and types of sensors and actuators used. The integrated systems were analyzed based on their primary functions and categorized into three groups: i) actuator‐assisted sensors for intelligent detection, ii) sensor‐assisted actuators for intelligent movement, and iii) sensor‐actuator interactive devices for a hybrid of intelligent detection and movement. In each section, we concentrate on the limitations of the conventional soft devices and the reasoning of the assisting sensors or actuators employed in the integrated devices for advanced intelligent systems, followed by a discussion of representative examples of each type with their working characteristics and diverse niche applications, such as adaptive biosensors on catheters,^[^ ^33^, ^35^ ^]^ adaptive moving robots,^[^ ^39^, ^41^, ^42^, ^43^, ^44^ ^]^ and prosthetic hands.^[^ ^48^, ^49^, ^53^, ^54^ ^]^ Finally, we discuss existing challenges and several promising strategies to resolve them and present an outlook for soft intelligent systems.

## 2 Types of Sensor–Actuator‐Integrated Systems

**Figure 1:**
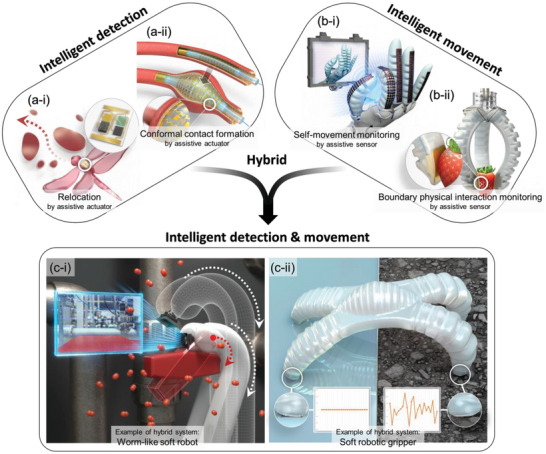
Types of sensor–actuator hybrid soft systems. a) Intelligent detection of actuator‐assisted sensors. i) Sensor is relocated to multiple target positions with delicate maneuvering by actuators. ii) Conformal contact with a target site using appropriate expansion of the soft substrate enhances the quality of sensor data. b) Intelligent movement of sensor‐assisted actuators. i) Integration of sensors on morphing actuators enabling self‐movement monitoring for accurate motion control. ii) Boundary physical interaction information, including contact pressure and force, can enable actuators to safely interact with surrounding objects. c) Combination of intelligent detection and movement. Intelligent detection and movement are both required in a combined manner to perform complex missions effectively. i) A worm‐like soft robot would require sensing capabilities to detect a gas leak and proprioceptive information to approach a target valve and turn it off. ii) An environment‐sensing soft robot may require sensing of the surface properties to adjust its gait pattern for faster and safer locomotion.

Integrated sensor and actuator systems can be categorized into three distinct types. Section 2.1 introduces the concept of intelligent detection using actuator‐assisted sensors, which have a wide range of practical applications. The integration of actuators enables the sensor to be actively repositioned and morphed, enhancing the quality and quantity of the sensor's output (**Figure** **1**a). Section 2.2 presents the idea of sensor‐assisted actuators, where proprioceptive information, such as geometrical deformation and boundary physical interaction, is obtained through integrated‐sensor data, leading to intelligent movement (Figure 1b). Finally, Section 2.3 describes the integration of intelligent detection and movement to perform complex missions. For instance, a gas sensor on a robot may be used to detect gas leakage, and the robot carefully moves toward the leakage point to turn off the valve using proprioception (Figure 1c(i)).^[^ ^46^ ^]^ Another example is an environmental monitoring robot that recognizes surface properties and adjusts its gait control parameters to sweep a region for multipoint measurements (Figure 1c(ii)).^[^ ^55^ ^]^

### 2.1 Actuator‐assisted Sensors for Intelligent Detection

**Figure 2:**
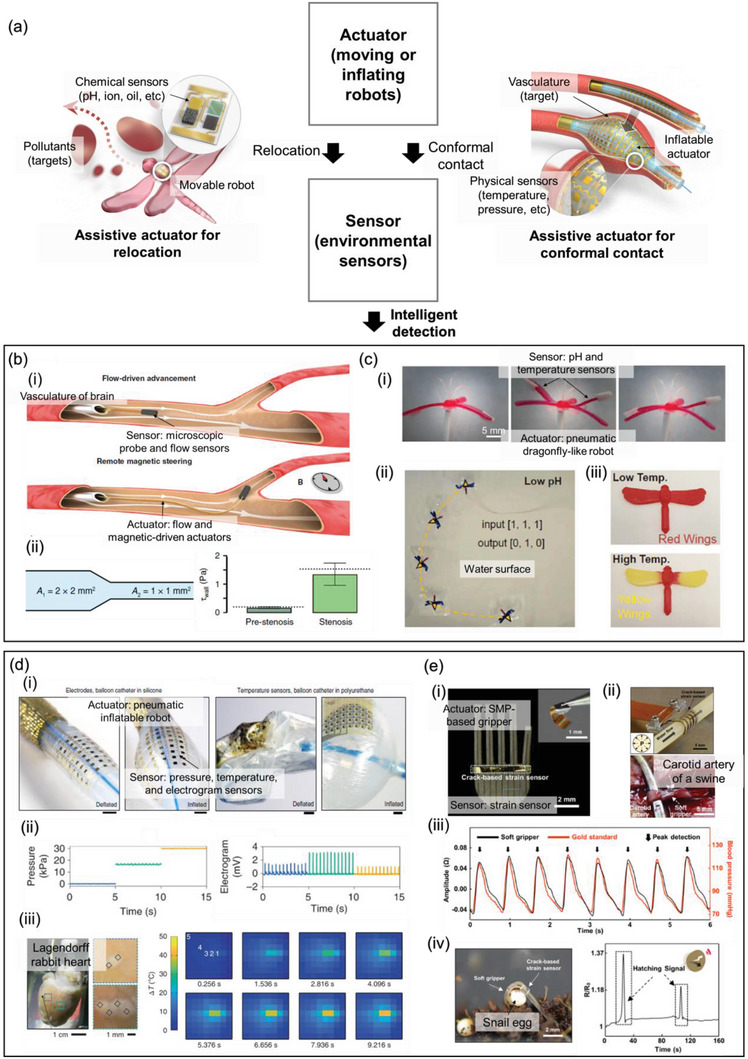
Intelligent detection based on actuator‐assisted sensors. a) Schematic illustrations and flowchart of the interaction mechanism and examples of the actuator‐assisted sensors. b) Intelligent flow sensing inside a vascular network. i) Flexible flow sensor is maneuvered to multiple target positions inside narrow and tortuous vascular network assisted by the fluid flow and applied magnetic field. ii) It performs on‐the‐fly measurement of wall shear stress to identify stenotic areas. Reproduced under the terms of the CC‐BY‐4.0 license.^[^ ^29^ ^]^ Copyright 2020, The Authors, Published by Springer Nature Limited. c) Soft robot with the shape of a dragonfly. i) It can steer on the water surface to identify the (ii) pH and iii) temperature, covering a vast searching area. Reproduced under the terms of the CC‐BY‐4.0 license.^[^ ^28^ ^]^ Copyright 2021, The Authors, Published by WILEY‐VCH Verlag GmbH & Co. KGaA, Weinheim. d) Inflatable soft catheter with intelligent detection. i) Catheter with a sensor array can inflate for conformal contact with target sites. Scale bars are 500 µm. ii) Appropriate contact force leads to a high signal‐to‐noise ratio. iii) Spatiotemporal mapping of temperature during a surgical procedure on a rabbit heart. Reproduced with permission.^[^ ^33^ ^]^ Copyright 2020, Springer Nature Limited. e) A multifunctional soft gripper to measure vital signals. i) Soft gripper consists of shape memory polymers with resistive strain sensors on top. (ii) Gripper can grab various vessels for conformal contact iii) to measure pulse signal. iv) Soft gripper can also conformally grab a snail egg to monitor the hatching process. Reproduced with permission.^[^ ^34^ ^]^ Copyright 2021, The American Association for the Advancement of Science.

For intelligent detection, sensors should identify locations of interest and adapt to the local environment. Without actuators, sensors cannot effectively manipulate or control the environment around them, which limits their ability to perform diverse and complex measurements once deployed. However, the integration of actuators and sensors enables mobility and adaptability through dynamic repositioning and morphing, allowing for more versatile and effective measurement capabilities. Actuator‐assisted sensors are systems in which actuators support sensors to improve the quality and quantity of measurements. In soft systems, sensors are assisted by actuators via two primary approaches, as shown in **Figure** **2**a. First, actuators facilitate the relocation of sensors. Based on diverse propulsion and translation mechanisms, including pneumatic and magnetic actuation, sensors can be repositioned at multiple locations to extract data from diverse sites. This is beneficial when intricate path‐following is required or a large area must be inspected. Second, actuators facilitate conformal contact with target surfaces. In nature, objects typically exhibit complex shapes with concavity, convexity, and roughness. Importantly, soft systems can be deformed to various shapes to conform to a variety of complex shapes of target objects. Therefore, expandable actuators and soft grippers are typically suitable for achieving conformal contact with target objects. With these additional capabilities, actuator‐assisted sensors in soft systems are often utilized for internal body monitoring and soft robots with sensing purposes.

The ability to relocate sensors enables a single device to perform measurements at multiple locations.^[^ ^56^, ^57^, ^58^ ^]^ For internal body monitoring, rendering the system soft and small, similar to body parts, is necessary for user comfort and reduces the risk of damage to surrounding tissues or organs. In such cases, it is necessary to insert sensors into the body, and multiple locations must often be examined to detect abnormalities. Sakar et al. developed an endovascular microscopic probe that can be steered in the complex vasculature of the brain.^[^ ^56^ ^]^ Delicate navigation through the cerebral vasculature is extremely challenging because of the catastrophic consequences of possible tissue damage and the bulkiness of existing tools. A microprobe has been fabricated based on a flexible flow sensor on a flexible and lightweight tether connected to a magnetic head; the viscous stress and pressure of surrounding blood flow have been utilized to maneuver the probe to target positions (Figure 2b(i)). In a single pathway, the device can follow tortuous vascular trajectories without mechanical instability owing to the controlled tension of the tether. In bifurcation, a magnetic field is applied to provide the appropriate torque on the magnetic tip for proper path selection. Using flow‐driven navigation, an on‐the‐fly measurement of wall shear stress was performed to detect the stenotic area, which is the region prone to clogging due to an abrupt decrease in the cross‐sectional area (Figure 2b(ii)).

Additionally, sensors can be combined with soft robots for relocation. Soft robots have multiple advantages including adaptable shapes, safe interactions with humans, lightweight, and resilience.^[^ ^59^ ^]^ Varghese et al. presented a pneumatically actuated soft robot that can be maneuvered on a water surface to characterize various parameters in the water.^[^ ^57^ ^]^ The shape of the robot resembles that of a dragonfly with two wings on each side. The hindwings can be bent upward under hydraulic control, enabling the control of the propulsion direction (Figure 2c(i)). By gliding on a water surface with selective propulsion from each side, the robot can cover a vast area and easily follow complex pathways to circumvent obstacles. They coated a hydrogel with pH‐responsive healing properties on the forewings and hindwings (Figure 2c(ii)). Because the hydrogel welds the forewings and hindwings at a low pH, the robot exhibited distinctive patterns of automotive motion depending on the pH level. By further coating the robot with a thermochromic pigment, the temperature of the water at multiple sites could be easily monitored (Figure 2c(iii)).

Sensors with expandable actuators achieve conformal contact with target objects for accurate measurements.^[^ ^60^, ^61^, ^62^ ^]^ Conformal contact is likely to increase the signal‐to‐noise ratio of the measurement, thereby enabling the monitoring of subtle signals. Rogers et al. developed an inflatable soft catheter integrated with sensor arrays for cardiac surgery (Figure 2d(i)).^[^ ^60^ ^]^ Sensor arrays for the spatiotemporal mapping of pressure, temperature, and electrograms were fabricated with a serpentine pattern for stretchability and transferred to a balloon catheter. After the device was placed in an appropriate position, such as the endocardium, the balloon was inflated to ensure conformal contact between the sensor array and target surface using pressure data, which ensured a high signal amplitude (Figure 2d(ii)). In an ex vivo study on a Lagendorff rabbit heart, the transition from regular to irregular heartbeats and the temperature distribution of the lesion and nearby surface were monitored during surgical procedures (Figure 2d(iii)). Obtaining a precise signal through conformal contact enhances the operator's performance and provides valuable information on the operation's progress.

Soft grippers can be used to achieve conformal contact with sensors.^[^ ^63^, ^64^ ^]^ When soft grippers are actuated, their grip follows the shape of the target object owing to their flexibility and stretchability. Han et al. developed a soft gripper based on a shape‐memory polymer (SMP) with strain sensors to monitor biosignals (Figure 2e(i)).^[^ ^63^ ^]^ The gripper was designed to enable a conformal grip by utilizing thermal actuation through Joule heating, allowing it to grasp multiple objects accurately. As shown in Figure 2e(ii,iii), the gripper grasps a carotid artery of a swine for in vivo measurement of blood flow with a crack‐based resistive strain sensor. Moreover, a snail egg was embraced by the gripper, and the hatching signal was monitored (Figure 2e(iv)). Without conformal contact with the gripper, the strain sensor would not have been able to measure a signal with a high signal‐to‐noise ratio.

In summary, intelligent detection was realized when sensors were assisted by actuators. Actuator‐assisted sensors can reposition themselves to perform measurements at multiple sites or deform their shapes to match the target objects for conformal contact. Although applications including internal body monitoring and soft robots have been introduced, intelligent detection can be extended to diverse fields if a wider variety of actuating and sensing mechanisms can be combined.

### 2.2 Sensor‐Assisted Actuators for Intelligent Movement

**Figure 3:**
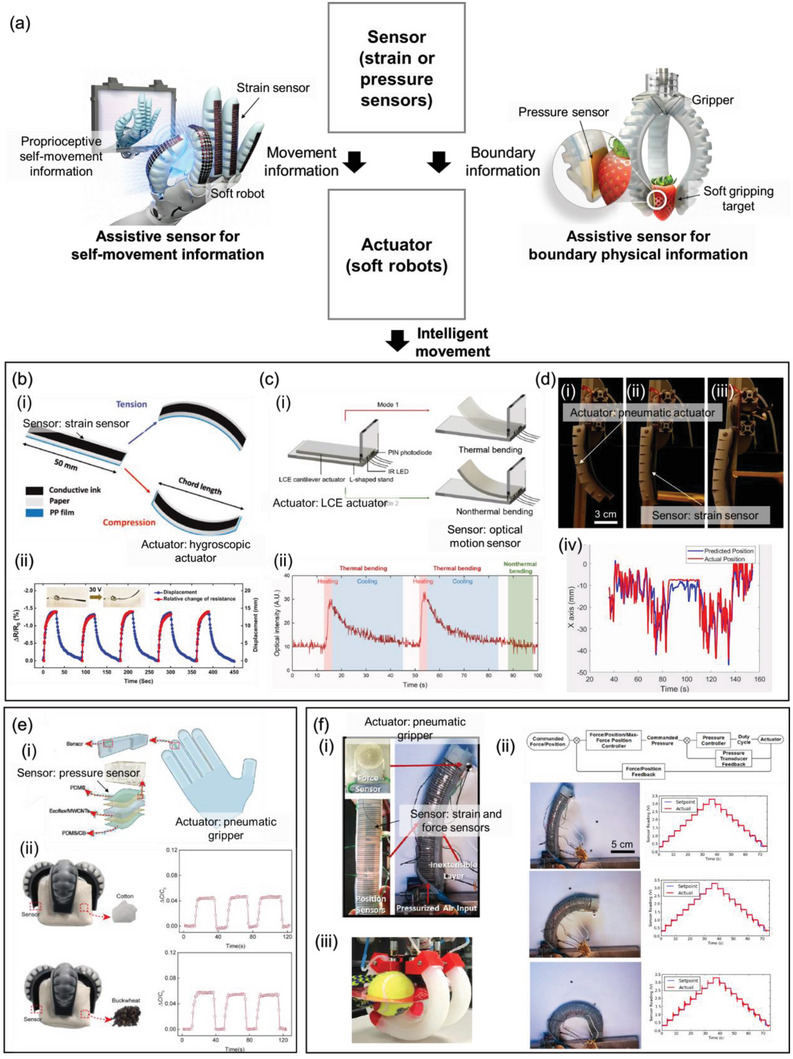
Intelligent movement using sensor‐assisted actuators. a) Schematic illustrations and flowchart of the interaction mechanism and examples of the sensor‐assisted actuators. b) A paper actuator with self‐movement sensing. i) The conductive ink on the paper experiences a change in resistance due to the bending caused by electrothermal actuation. ii) The change in resistance reflects the geometrical deformation of the paper actuator. Reproduced under the terms of the CC‐BY‐4.0 license.^[^ ^65^ ^]^ Copyright 2018, The Authors, Published by WILEY‐VCH Verlag GmbH & Co. KGaA, Weinheim. c) A thermomechanical liquid crystal elastomer (LCE) with self‐movement sensing. i) The actuator can be bent through either thermal or nonthermal means. ii) The phase shift of LCE with temperature change leads to a change in transparency. A pair of light‐emitting diode and a photodiode is utilized to monitor the bending due to the thermal actuation. Reproduced under the terms of the CC‐BY‐4.0 license.^[^ ^66^ ^]^ Copyright 2021, The Authors, Published by The American Association for the Advancement of Science. d) A soft pneumatic actuator with proprioception based on a recurrent neural network. It is placed under three different conditions at random: i) unconstrained, ii) tip‐constrained, and iii) center‐constrained. iv) Using data from the three built‐in strain sensors, the position of the tip of the actuator can be inferred, regardless of the constraining condition. Reproduced with permission.^[^ ^43^ ^]^ Copyright 2021, The American Association for the Advancement of Science. e) A soft pneumatic gripper that can sense the contact pressure. i) At the tip of each finger, a capacitive pressure sensor is placed. ii) Actuator needs to hold a heavier package with higher holding pressure to avoid slip from the actuator. Reproduced with permission.^[^ ^39^ ^]^ Copyright 2021, Elsevier Ltd. f) A soft pneumatic actuator with sensing capabilities of both self‐movement and force interaction. i) At the tip of the soft pneumatic gripper, a resistive force sensor is placed, while position sensors are located on the extending side of the actuator. ii) Feedback control of the actuator enables desired motion and force interaction with the surroundings. iii) A gripper featuring these fingers can effectively manipulate common objects. Reproduced with permission.^[^ ^41^ ^]^ Copyright 2016, IEEE.

Actuators need proprioception, which refers to their awareness of their current state in terms of internal geometry and force interaction, to enable intelligent movement. For an actuator to achieve certain actions such as grasping an object, the information of its geometrical state and force interaction with the environment is vital for accurate motion control. To achieve the desired action, closed‐loop (i.e., feedback) control necessitates sensor data to provide feedback on the current state of actuators to the controller, allowing for continuous modification of the control input. Compared with open‐loop control, which is a control method without sensor data, feedback control is more robust to disturbances such as changes in constraining conditions. Hence, sensor‐assisted actuators are systems in which sensors provide proprioceptive information about the actuators to achieve a desired action more precisely, as shown in **Figure** **3**a. Various soft actuators that operate with soft strain and force sensors to obtain accurate and reliable proprioceptive information have been reported. Depending on the type of proprioceptive information, they can be classified into three groups: self‐movement, boundary physical interaction, and a combination of the two.

Soft actuators can be assisted by proprioceptive strain sensors to accurately follow motion commands.^[^ ^43^, ^44^, ^65^, ^66^, ^67^, ^68^, ^69^, ^70^, ^71^, ^72^ ^]^ Film‐based bending actuators often employ a mismatch between the thermal or hygroscopic expansions of two materials as the bending mechanism. Amjadi et al. incorporated a resistive strain sensor into a bending actuator based on a film, which allowed for the provision of thermal energy through Joule heating and bending information (Figure 3b).^[^ ^65^ ^]^ As the film was heated, the actuator was bent under hygroscopic expansion on one side and the corresponding contraction on the other side. Under bending motion, the strain sensor was stretched to increase resistance due to the growing disconnection between conductive regions. Because resistance is often highly affected by temperature, the strain data must be decoupled from the temperature. They mixed graphite and carbon nanotubes, which have positive and negative thermal coefficients of resistance (TCR), respectively, in an appropriate ratio to obtain a hybrid film with nearly zero TCR. Li et al. fabricated a film‐based bending actuator using liquid‐crystal elastomers (LCE) for optomechanical self‐movement sensing (Figure 3c).^[^ ^66^ ^]^ The bending motion could be achieved by controlling the temperature of the film owing to the asymmetric photocuring of the LCE. As the transparency of the LCE increases with temperature from the phase transition, the bending state from thermal actuation can be measured using a pair of light‐emitting diode (LED) and photodiode (PD). As the temperature increases, the LCE becomes more bent and transparent, leading to less light scattering from the LED and higher light intensity detected by the PD. In this case, the optical data was influenced by temperature, allowing for a selective response to bending caused by thermal actuation only. While studies have demonstrated successful self‐movement sensing of soft actuators, movement in more general environments may be constrained by obstacles such as trees or rocks. Thuruthel et al. addressed this issue using a recurrent neural network (RNN).^[^ ^43^ ^]^ As illustrated in Figure 3d, a pneumatic actuator equipped with three internal strain sensors was subjected to one of the following conditions: unconstrained, constrained at the tip, or constrained at the middle of the body. Subsequently, the RNN was trained to predict the tip position of the actuator under a bending motion, with the pneumatic pressure and impedance values of the three resistive sensors as inputs. After training, the RNN could infer the motion of the pneumatic actuator in real time, even with the random addition of constraints. This demonstrates the possibility of accurately controlling soft actuators in diverse environments, even when obstacles hinder their motions.

Information regarding the physical interaction of the boundary with the surroundings can also assist in the motion of actuators. Without force information, the actuator may fail to achieve the desired motion.^[^ ^39^, ^45^, ^73^ ^]^ For example, a robotic gripper must grab objects with an appropriate force by considering their fragility and mass to avoid dropping or crushing them. Zou et al. developed a pneumatic soft gripper with porous pressure sensors on the tip to monitor contact forces (Figure 3e(i)].^[^ ^39^ ^]^ When the gripper comes in contact with an object, the capacitive pressure sensor located at the tip gets compressed due to the contact force. This compression causes an increase in capacitance as there is a decrease in distance and an increase in the dielectric constant between the two parallel electrodes of the sensor. The robotic gripper could grab sandbags of different materials (Figure 3e(ii)). As a bag with buckwheat is heavier than that with cotton, the gripper had to squeeze the bag with a greater contact force (Δ*C*/*C*_0_ = 0.04 for cotton and 0.06 for buckwheat) to successfully pick up the heavier bag. Force‐controlled soft grippers can be directly utilized in food production lines to pick and place delicate fruits and vegetables of various sizes and masses through simple motions.

Soft actuators require information on both self‐movement and boundary physical interactions for complex tasks such as those required of household robots.^[^ ^41^ ^]^ Morrow et al. added three resistive strain sensors on the extensible side of soft fingers for self‐movement measurements and a resistive force sensor on the tip for boundary force measurements (Figure 3f(i)).^[^ ^41^ ^]^ Similar to previous studies, the deformation of microchannels filled with liquid metal due to bending deformation or contact force leads to a change in resistance. Using the information on the current state of the actuator, an appropriate command control input was calculated and applied to reduce the deviation between the current and desired states. Using this feedback control, the actuator could exert the desired force in various bending states created by different constraints (Figure 3f(ii)). Additionally, a robotic hand consisting of four pneumatic actuators was developed and successfully used to pick up 27 different objects, including a book, a tennis ball, and a trash can. The position of the fingers was controlled to fit each object, while force control was used to grip it securely without causing any damage.

The intelligent motion of actuators requires sensor data for self‐movement and physical boundary interactions. By using proprioceptive information, actuators can deform to desired shapes and apply appropriate forces to accomplish complicated motion tasks. For practical applications, the reliable working of the proposed sensor‐actuator hybrid in generalized environments with diverse constraints should be considered and addressed in future studies.

### 2.3 Sensor‐Actuator Interactive Devices for Hybrid of Intelligent Detection and Movement

Intelligent detection and movement can significantly enhance the quality and reliability of motion and sensing, respectively. However, to achieve complex missions, a combination of intelligent detection and movement is necessary. For instance, in internal body monitoring, an intelligent device should travel safely to sites of interest with proprioception, collect reliable data based on conformal contact, and provide immediate treatment for any abnormalities detected. As a complex mission involves multiple steps of intelligent detection and movement, the development of sensor‐actuator interactive devices that can perform both is a promising area of research. Despite the limited number of studies in this field because of the still‐evolving stages of intelligent detection and movement, we introduce two types of devices capable of both intelligent detection and movement to complete complex missions: worm‐like robots with visual feedback^[^ ^46^, ^47^ ^]^ and hand‐like robots with tactile sensation.^[^ ^48^, ^49^, ^53^, ^54^ ^]^

**Figure 4:**
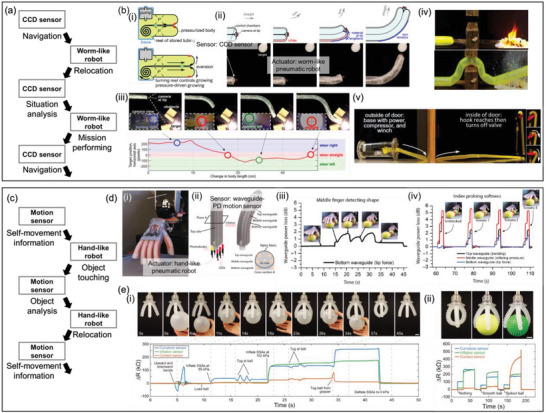
Hybrid system comprising both intelligent detection and movement. a) Flowchart of the interaction between the sensor and actuator in a worm‐like robot. b) Growing soft robots. i) A schematic illustration of a growing mechanism for navigation. Turning the reel and controlling the pressure leads to the eversion of the membrane, leading to the growth of the robot. ii,iii) Visual sensing enables the proprioception and path planning to iv) detect and extinguish fire, or v) approach to and turn off a valve to stop gas leakage. Reproduced with permission.^[^ ^46^ ^]^ Copyright 2017, The American Association for the Advancement of Science. c) Flowchart of the interaction between the sensor and actuator in a hand‐like robot. d) Optoelectronically innervated soft prosthetic hand. i) Prosthetic hand comprising ii) fingers with proprioceptive sensing based on optical data. iii) It can stroke the surface to locate multiple tomatoes and iv) reposition the hand to measure the softness by pressing them individually for a ripeness test. Reproduced with permission.^[^ ^48^ ^]^ Copyright 2016, The American Association for the Advancement of Science. e) Soft somatosensitive gripper. i) Its interaction with a ball is reflected in the three‐sensor data. ii) Furthermore, the gripper can conformally contact objects to distinguish different textures. Scale bars are 20 mm. Reproduced with permission.^[^ ^49^ ^]^ Copyright 2018, WILEY‐VCH Verlag GmbH & Co. KGaA, Weinheim.

Worm‐like robots with visual feedback are capable of both intelligent detection and movement to accomplish complex missions, including turning off valves in disaster scenes and performing biopsies through a highly elastic multibranched pipe, as shown in **Figure** **4**a. Hawkes et al. proposed a growing soft worm‐like robot, termed a vine robot, with intelligent detection and movement capabilities.^[^ ^46^ ^]^ It comprised multiple plastic membranes that were selectively pressurized to grow in a certain direction using a controlled reel (Figure 4b(i,ii)). By mounting a camera on the front tip, the robot could analyze real‐time images to determine its growth path and inspect the site to determine the next action (Figure 4b(iii)). For example, the robot detected and grew toward a fire to safely extinguish it (Figure 4b(iv)), turned off a valve inside a room with a hazardous gas leakage, and started to grow outside the door (Figure 4b(v)). The robot could be scaled down to function as a catheter for ablation inside the brain ventricle. Zhang et al. developed a worm‐like soft robot to operate in complicated tubular environments.^[^ ^47^ ^]^ Their pneumatic soft robot consisted of three modules: balloon‐like expanding head and end, and longitudinally extending center. The robot could move forward by selective anchoring of the head or end and sequential lengthening and shortening motion of the central module. Moreover, the direction of movement for the robot was customizable due to the presence of a central module that contained three chambers arranged in parallel. By selectively extending these chambers, the robot could be bent in a particular direction. They mounted a micro charge‐coupled device (CCD), an electromagnetic‐tracking sensor, and biopsy forceps as proprioception and measurement devices. The robot could navigate through multi‐branched pipes based on local information from the CCD and global position data from the electromagnetic sensor and perform a biopsy from multiple sites. Overall, worm‐like soft robots could actuate desired motions using sensor data and reposition sensors at multiple sites for inspection and measurement.

Hand‐like robots with tactile sensation have also been developed to perform complex missions with multiple sub‐tasks using a combination of intelligent detection and motion, as shown in Figure 4c. For instance, Zhao et al. fabricated an optoelectrical innervated soft prosthetic hand for multipurpose applications (Figure 4d(i)).^[^ ^48^ ^]^ Each finger comprised a pneumatic soft actuator with three innervated stretchable waveguides, the ends of which were placed in an LED–PD pair (Figure 4d(ii)). The deformation of the finger actuator (bending, pressing, and elongating) was encoded by the intensity variation of the data from the three PDs. They demonstrated the use of the prosthetic hand to recognize the position and number of tomatoes and identify ripe ones. First, the hand gently stroked over the objects. During motion, the finger could deform freely, following the shape of the tomatoes, and the angle and height of the hand were fixed. Using a known stroke speed and finger deformation, the contour of the stroke path could be obtained (Figure 4d(iii)). Then, the hand pressed each tomato at three different pressures to measure the degree of softness. Because unripe tomatoes are stiffer than ripe ones, the deformation of the finger is different under the same pneumatic actuation. Therefore, the robot could both locate and distinguish between ripe and unripe tomatoes (Figure 4d(iv)). Truby et al. developed a soft robotic hand equipped with three resistive sensors that can detect the curvature, inflation, and contact pressure of each finger.^[^ ^49^ ^]^ The three sensors collected data that demonstrated the interaction between the hand and a ball, as well as a human's interaction with the hand, providing proprioceptive information (Figure 4e(i)). The data revealed that the ball was grasped at two different actuation pressures and was forcefully released at the end, all of which were distinguishable from the data. The hand was also capable of identifying the textures of the grasped objects. Using the proprioceptive information, the gripper's tip could be seamlessly brought into contact with objects of varying textures, and the contact sensor produced different outputs when encountering smooth and spiked balls (Figure 4e(ii)). Soft robotic hands are a highly researched area in soft robotics, with numerous studies indicating their capacity to carry out intricate tasks that necessitate a blend of intelligent detection and motion.^[^ ^53^, ^54^ ^]^

Complex missions typically involve multiple steps including intelligent detection and movement. For a specific mission, a robotic system must solve specific sets of problems that require interaction with the environment and inspection of multiple targets to make informed decisions regarding the next action. Such interactive systems can replace and also surpass human labor, as they can be designed to fit a range of form factors. Possible applications include household, surgical, and rescue robots in disaster scenarios. The integration of various sensors will further extend the applications of interactive soft systems.

## 3 Existing Challenges and Perspectives

Sensor–actuator hybrid soft systems are being applied synergistically in various fields, as explained in this review, and their feasibility for intelligent applications has already been proven. However, these studies are still in the initial stages, and there is a need for substantial improvements that can significantly contribute to their development.

First, the individual technology levels of soft sensors and soft actuators must be improved. Soft sensors and soft actuators currently have a limited range of applications, making it difficult to select the ideal option for a specific purpose. For instance, a variety of rigid pressure sensors with diverse morphological and functional features have been developed, allowing them to be easily employed for specific purposes. However, soft pressure sensors face various limitations, such as material constraints (usually polymer‐based) and fabrication methods, which restrict their development for specific applications. Thus, some systems partially adopt rigid elements, while the entire system is flexible. For example, Han et al. proposed a smart catheter that can monitor the temperature and pressure distributions of surrounding vessels.^[^ ^33^ ^]^ Because flexible pressure sensors cannot easily be arrayed with high resolution using the current technology, a rigid and miniaturized pressure sensor array is integrated into a smart catheter. Although fully soft sensor‐actuator hybrid systems cannot easily be achieved owing to technological limitations, the overall research trend of sensor–actuator hybrid systems is toward fully soft systems. Therefore, we anticipate that the development of hybrid systems with rigid components will continue to advance, eventually falling under the scope of this review.

Second, interference between soft sensors and actuators inevitably occurs and should be compensated. For instance, the signal of a sensor is usually generated by a change in its electrical properties (e.g., a change in resistance). However, soft actuators are driven by changes in various physical parameters, such as temperature, magnetic field, and electric field, which significantly interfere with the electrical signal. Therefore, the integration of the soft sensors and actuators is inevitably accompanied by various physical/electrical crosstalks, thus lowering the accuracy of the soft sensors. In rigid systems, these issues can be addressed using various pre‐existing packaging and shielding methods. However, the available materials and mechanisms for soft systems are limited, highlighting the need for improvements. Another possible solution is the further development of software methods. In line with the recent development process of sensors, fast and high‐performance signal processing methods such as deep learning have recently been developed.^[^ ^74^, ^75^, ^76^, ^77^, ^78^ ^]^ When the results of these advances are applied to the sensor‐actuator hybrid system, it is believed that the performance of the overall system can be improved not only by solving the crosstalk between components but also by advancing the fundamental deploying technology of the overall system.

Third, there is a lack of suitable mechanisms for soft actuators that can be utilized in sensor‐actuator hybrid systems. In most studies, pneumatic actuation has been adopted as the primary actuation mechanism for the following reasons. Sensors in soft sensor‐actuator hybrid systems are positioned on the actuators, making them susceptible to the physical/electrical signals that generate the actuator's movement. This is not a concern for pneumatic actuation, as these signals can be readily eliminated. In addition, the fabrication methods of the pneumatic actuator are simpler than other actuating mechanisms. Although the utilization of pneumatic actuators is advantageous for the abovementioned reasons, it is important to explore various types of soft actuators depending on the specific applications. For instance, pneumatic actuation may not be suitable for use in extreme pressure conditions, such as those involving extremely high pressure or a vacuum state that cannot be easily controlled. In such cases, other mechanisms, such as those employed in electrothermal actuators, may be more appropriate. Thus, there is a need to expand the range of mechanisms used for soft actuators in sensor‐actuator hybrid systems, such as including devices that utilize electrothermal actuators.^[^ ^79^ ^]^ This improvement has the potential to significantly broaden the range of fields in which soft sensor‐actuator hybrid systems can be applied.

## 4 Conclusion

Soft sensors and actuators have demonstrated their potential for use in numerous fields and have been the subject of active research. Previous studies have primarily focused on enhancing these areas, and as technology advances, integration and synergistic utilization of these fields in various applications have become possible. This review provided a detailed introduction to this trend by outlining three types of intelligent systems: intelligent detection (actuator‐assisted sensor), intelligent movement (sensor‐assisted actuator) systems, and a hybrid of intelligent detection and movement (sensor and actuator assist each other), as shown in **Table** **1**. While integration of these elements is still in the early stages, initial studies have demonstrated significant potential for intelligent usage of each component, reinforcing their original functions. Furthermore, resolving the abovementioned challenges is expected to enhance this potential further. For example, the current trend toward prosthetics and human‐machine interfacing has the potential to materialize through the sensor‐actuator hybrid system. Presently, the system's overall maturity remains lacking, making a direct replacement of the existing rigid prosthetic systems challenging. Nonetheless, progress is anticipated to empower the development of pliable prosthetic elements like hands and fingers. This advancement will serve as a robust substitute for advanced prosthetic systems, showcasing enhanced interaction with biological tissue and superior capabilities.

**Table 1:** Representative Examples of Sensor‐Actuator Hybrid Systems for Soft Intelligent Systems.

Reference	System type	Individual components		Characteristics (including advantages and disadvantages)	Applications
		Sensor type	Actuator type		
[29]	Actuator‐assisted sensors for intelligent detection	Flow sensing	Magnetic actuation	On‐the‐fly measurement of wall shear stress; requires delicate magnetic control	Stenotic region detection in avascular network
[28]	Actuator‐assisted sensors for intelligent detection	Temperature and pH sensing	Pneumatic thrust	Maneuverability on water surface to cover vast area; requires tethering	Water acidification and temperature detection
[33]	Actuator‐assisted sensors for intelligent detection	Temperature and pressure distribution sensing	Pneumatic expansion	Conformal contact with target object to increase signal‐to‐noise ratio; non‐uniform contact pressure for objects with complex shapes	Operation monitoring
[34]	Actuator‐assisted sensors for intelligent detection	Strain sensing	Thermally actuated shape memory polymer	Ability to gently grab a vessel to monitor the flow inside; contact with a heated surface	Hatching/vital signal monitoring
[65]	Sensor‐assisted actuators for intelligent movement	Strain sensing	Electrothermally induced hygroscopic expansion	Temperature decoupled bending monitoring; weak actuation force	–
[66]	Sensor‐assisted actuators for intelligent movement	Temperature sensing	Thermal actuation	Selective detection of thermally induced bending; weak actuation force	–
[43]	Sensor‐assisted actuators for intelligent movement	Strain sensing	Pneumatic expansion	Ability to infer tip position regardless of the addition of motion constraints; considered point constraints only	–
[36]	Sensor‐assisted actuators for intelligent movement	Pressure sensing	Pneumatic expansion	Delicate contact based on pressure monitoring; neglect of shear force	Holding up objects of different weights
[41]	Sensor‐assisted actuators for intelligent movement	Strain/force sensing	Pneumatic expansion	Feedback control of the motion and action; overshooting responses	Picking up ordinary objects
[47]	Hybrid intelligent system	Vision sensing, position sensing	Pneumatic expansion	Ability to navigate in a multi‐branched pipe to perform biopsy; slow motion	Pipe monitoring
[46]	Hybrid intelligent system	Vision sensing	Pneumatic growth	Fast growing toward a target site; limited end effector	Closing a valve; extinguishing a fire
[48]	Hybrid intelligent system	Strain sensing	Pneumatic expansion	Softness detection,contour detection after a gentle stroke; translation motion by a rigid robotic arm	Location and ripeness determination of tomatoes
[49]	Hybrid intelligent system	Curvature/contact/inflation sensing	Pneumatic expansion	Texture detection, object handling capability; transient response at a constant high‐contact pressure	Interaction with a ball and texture detection

## Conflict of Interest

The authors declare no conflict of interest.
